# Performance assessment of computational tools to detect microsatellite instability

**DOI:** 10.1093/bib/bbae390

**Published:** 2024-08-12

**Authors:** Harrison Anthony, Cathal Seoighe

**Affiliations:** School of Mathematical and Statistical Sciences, University of Galway, Galway H91 TK33, Ireland; The SFI Centre for Research Training in Genomics Data Science, Galway D02 FX65, Ireland; School of Mathematical and Statistical Sciences, University of Galway, Galway H91 TK33, Ireland; The SFI Centre for Research Training in Genomics Data Science, Galway D02 FX65, Ireland

**Keywords:** cancer biomarker, benchmarking, microsatellite instability

## Abstract

Microsatellite instability (MSI) is a phenomenon seen in several cancer types, which can be used as a biomarker to help guide immune checkpoint inhibitor treatment. To facilitate this, researchers have developed computational tools to categorize samples as having high microsatellite instability, or as being microsatellite stable using next-generation sequencing data. Most of these tools were published with unclear scope and usage, and they have yet to be independently benchmarked. To address these issues, we assessed the performance of eight leading MSI tools across several unique datasets that encompass a wide variety of sequencing methods. While we were able to replicate the original findings of each tool on whole exome sequencing data, most tools had worse receiver operating characteristic and precision-recall area under the curve values on whole genome sequencing data. We also found that they lacked agreement with one another and with commercial MSI software on gene panel data, and that optimal threshold cut-offs vary by sequencing type. Lastly, we tested tools made specifically for RNA sequencing data and found they were outperformed by tools designed for use with DNA sequencing data. Out of all, two tools (MSIsensor2, MANTIS) performed well across nearly all datasets, but when all datasets were combined, their precision decreased. Our results caution that MSI tools can have much lower performance on datasets other than those on which they were originally evaluated, and in the case of RNA sequencing tools, can even perform poorly on the type of data for which they were created.

## Introduction

Microsatellite instability (MSI) is a phenomenon characterized by the accumulation of insertions and deletions (indels) in microsatellite regions found throughout the genome [1]. First described in hereditary non-polyposis colorectal cancer [2, 3], it has now been observed across multiple cancer types [4] and in both sporadic as well as familial cancers [5]. While it has yet to be demonstrated in a laboratory setting exactly how MSI arises, the predominant hypothesis is that defects in the DNA mismatch repair pathway can cause an increase in the number of indels at microsatellite sites [6]. This marked increase in the rate of indels is the primary characteristic of MSI and how it is primarily identified. The identification of MSI is important because cancers with high microsatellite instability (MSI-H) can be good candidates for immune checkpoint inhibitor treatment [7]. Historically, MSI-H has been inferred from the PCR amplification of five microsatellite markers [8, 9]. However, next-generation sequencing (NGS) allows the MSI status to be inferred using a larger number of loci and enables the determination of the MSI status to be incorporated into a comprehensive genome profiling pipeline [10].

A variety of computational tools and algorithms have been developed to determine the MSI status from NGS data. MSI tools here refers to programs that are available for use with NGS data and that can be quickly incorporated into a research or clinical bioinformatics pipeline whereas algorithms would require additional work before they could be used with NGS data. Tools differ primarily in the type of sequencing data used to determine the MSI status and use either DNA, RNA, or multi-omic sequencing data. The majority of tools use DNA sequencing data to compare the indels in microsatellites between a tumor and paired-normal sample. One example, and one of the first MSI tools, MSIsensor [11], uses a relatively simple algorithm that compares tumor and normal k-mer read length counts at each microsatellite and identifies microsatellite sites as unstable if they are significantly different in a χ^2^ test. Other tools compare the gene expression values from RNA sequencing data to pre-trained baseline expression values. PreMSIm is an example of a tool that predicts the MSI status by using a k-nearest neighbors classification algorithm based on the expression of 15 genes [12]. Multi-omic tools use more complex algorithms. One example is DeltaMSI [13], which distinguishes unstable loci using a machine learning model built with both immunohistochemistry (IHC) data and NGS data. Other researchers have already created exhaustive lists of these tools and described their methods in detail [14–16], but, in general, all MSI tools classify samples as having MSI-H or as being microsatellite stable (MSS). The output of all tools is a value representing the level of MSI present in a sample – typically reported as the proportion of microsatellite sites that are unstable. Lastly, a threshold is picked to distinguish MSI-H from MSS.

The authors of most tools provide a recommended threshold to distinguish MSI-H from MSS samples; however, the recommended settings and scope of MSI tools are sometimes unclear. For example, MSIsensor [11], was originally tested on whole exome sequencing (WXS) of 242 endometrial tissue samples from The Cancer Genome Atlas (TCGA). The latest version of MSIsensor comes with both WXS and whole genome sequencing (WGS) recommended settings despite having never been tested on WGS samples. This is also the case for the two successor tools MSIsensor-pro [17] and MSIsensor2 [18]. Two other widely used MSI tools, mSINGS [19] and MANTIS [20], both provide recommended thresholds to define MSI-H using WXS data but, like the other tools, it is not clear whether they are applicable to other sequencing types. This is also the case for a more recent tool, MSINGB [21], which does not require the user to set a threshold and simply outputs the status of the tumor sample. The only MSI tools that have been trained and tested on RNA sequencing data, PreMSIm [12] and MSIsensor-RNA [22], do not require the user to pick a threshold and, like MSINGB, will output the MSI status of a sample. They also indicate that they can be used with bulk and single-cell RNA sequencing, as well as microarray panels. However, there has not yet been an independent benchmarking of MSI NGS tools to verify the reported performance metrics of each tool and to determine the factors that affect those performance metrics.

Here we set out to address these issues by benchmarking the leading MSI tools on several datasets derived from different sequencing strategies. These included MSIsensor, which was among the first MSI tools to be created and which has also received FDA approval [11]; MSIsensor2 [18], a tool used to calculate an MSI score from the National Cancer Institute’s Genomic Data Commons (GDC); MSIsensor-pro [17], which claims to improve upon the original MSIsensor by including a unique tumor-only algorithm with higher accuracy; mSINGS [19], the first tumor-only MSI tool and a tool that is commonly used with gene panel data; another heavily cited, paired-normal tool, MANTIS [20]; a very recent tool that uses somatic variant information to classify samples, MSINGB [21]; and two tools made solely for use with RNA sequencing data, PreMSIm [12] and MSIsensor-RNA [22]. By measuring tool performance on data derived from a broad range of sequencing methods, we aimed to replicate the high published performance metrics of each tool and determine which tools work best across sequencing types. Our results shed light on tool performance under optimal and non-optimal conditions and potential shortcomings in the underlying algorithms used to classify samples as MSI-H versus MSS.

## Methods

### Datasets

All TCGA WXS, WGS, and RNA sequencing data was downloaded in BAM format from the GDC Data Portal (https://gdc-portal.nci.nih.gov/) (Table 1). All WXS and WGS samples were subset down to only microsatellite regions to help with storage and processing time. We were able to obtain PCR MSI status for a total of 852 WXS and 321 WGS paired tumor-normal samples as well as 825 tumor-only RNA sequencing samples from the Broad Institute Genome Data Analysis Center (https://gdac.broadinstitute.org). Our TCGA sample list is based on those used in another publication from Cortes-Ciriano et al. [23], which has already compiled a list of all TCGA cohorts that have matched PCR MSI status. For the purposes of binary classification (used by all MSI tools), we treated MSI-L samples as MSS.

**Table 1:** TCGA datasets

Project ID	Cancer	Sequencing	Number of Samples	Number of MSS	Number of MSI-H
COAD	Colon	WGS	56	46	10
ESCA	Esophageal	WGS	2	0	2
STAD	Stomach	WGS	136	107	29
UCEC	Uterine/endometrial	WGS	145	102	43
COAD	Colon	WXS	284	232	52
ESCA	Esophageal	WXS	3	0	3
READ	Rectum	WXS	3	0	3
STAD	Stomach	WXS	292	228	64
UCEC	Uterine/endometrial	WXS	268	196	72
COAD	Colon	RNA	280	230	50
ESCA	Esophageal	RNA	3	0	3
READ	Rectum	RNA	3	0	3
STAD	Stomach	RNA	272	213	59
UCEC	Uterine/Endometrial	RNA	268	196	72

The metadata for all TCGA datasets with paired MSI status (MSS is microsatellite stable, and MSI-H is high microsatellite instability). Whole genome sequencing and whole exome sequencing are abbreviated to WGS and WXS, respectively.

All gene panel samples were downloaded in FASTQ format from the Sequence Read Archive (SRA) (https://www.ncbi.nlm.nih.gov/sra). We aligned the raw SRA FASTQ files to the same human reference genome used by TCGA (GRCh38.p14). The alignment for all samples was done using BWA [24]. In total, we collected 142 TSO-500 gene panel samples uploaded as part of three separate studies [25–27]. We also used 191 samples from the Oncomine 161-marker gene panel [28] and 178 samples from a 6-mononucleotide repeat paired-normal panel [29]. The TSO-500 and Oncomine samples also have an MSI score given by their commercial software, the Illumina TSO-500 pipeline and MSIcall, respectively. We used a threshold cut-off of 15 for the TSO-500 pipeline based on the current pre-print [30]. For the Oncomine samples, only MSI status as determined by MSIcall was given by the authors of the publication [28].

The other additional sequencing datasets we used for testing included DNA and RNA sequencing datasets. They were collected and managed the same way as the gene panel samples and were also from the SRA. However, for the RNA sequencing data, we used a different alignment software, STAR [31], with the same GRCh38 reference genome, and then created gene count matrices with FeatureCounts [32]. Gene counts then underwent log_2_(n + 1) normalization for use with MSIsensor-RNA and then also scaled to be between 0 and 1 for PreMSIm. The MSI status for each of these datasets was determined with either PCR or IHC, or based on the fact that the samples were from MSI-H tumor cell lines (Table 2). These datasets comprised 29 tumor-only WXS samples [33], 21 paired-normal WXS samples (Bioproject: PRJNA727917), 34 End-seq samples [34], and 143 tumor-only bulk RNA sequencing samples [35] (Table 2).

**Table 2:** Additional non-TCGA datasets

Project ID	Cancer	Sequencing	Number of Samples	Number of MSS	Number of MSI-H
PRJNA629785	Colorectal	End-seq	34	7	27
PRJNA810563	Pan	6 Marker Panel	178	166	12
SRP008162	Prostate	T/O WXS	21	16	5
PRJNA727917	Colorectal	P/N WXS	21	0	21
PRJNA256024	Prostate	53 Marker Panel	43	30	13
PRJNA701182	Pan	161 Marker Panel	191	185	6
PRJNA841034	Gastric	TSO500	36	34	2
PRJEB57620	Male Breast	TSO500	14	14	0
PRJNA843231	Pan	TSO500	14	11	3
PRJNA748264	Colon	RNA	143	122	21

The metadata for all additional non-TCGA datasets with paired MSI status (MSS is microsatellite stable, and MSI-H is high microsatellite instability). The project ID is the searchable accession number for the Sequence Read Archive. MSI status establisher was the method used by the original publications to determine MSI status for each sample.

One tool, MSINGB, required extra data handling before it could be used. For TCGA WXS samples, Mutect2 VCF files were readily available and were downloaded from the GDC. For all other DNA sequencing datasets, we created VCF files following Mutect2 best practice settings [36]. It was run in paired-normal mode unless the sample lacked a paired-normal, then it was run with tumor-only mode. Lastly, we converted the VCF files to MAF format using vcf2maf [37].

### MSI tools and software settings

Eight MSI tools were evaluated in total. These were: MSIsensor [11], MSIsensor2 [18], MSIsensor-pro [17], mSINGS [19], MANTIS [20], MSINGB [21], PreMSIm [12], and MSIsensor-RNA [22] (Table 3). Each tool was run with author recommended settings, though MANTIS and mINSGS did not provide WGS recommendations. For MANTIS to work with WGS we had to lower the minimum locus quality to 15, the minimum average per-base read quality to 10, and the minimum coverage threshold to 10. Without these adjustments, MANTIS could not find usable microsatellite sites. We did not adjust any settings for mSINGS or MSINGB and applied the same settings used with WXS. This was done because there were no parameters to help correct for the difference in sequencing depth. As we expected the coverage of microsatellites to be low in RNA sequencing data, we ran MSIsensor2, MSIsensor-pro, mSINGS, with the same settings used with WGS data.

**Table 3:** MSI tool summaries

Tool	Original evaluation data	Algorithm used for MSI detection	Output (MSI score)	Recommended threshold	Requires paired normal
MSIsensor	242 endometrial TCGA WXS samples	χ^2^ test between tumor and normal read counts	Percent of unstable microsatellites	3.5	Yes
MSIsensor-pro	1532 pan-cancer TCGA WXS samples	Multinomial distribution model distinguishes MSI sites by comparing probability of polymerase slippage	Percent of unstable microsatellites	None	No
MSIsensor2	117 EGA samples and 10 TSO500 samples (TCGA also used but not numerically described)	Machine learning based (specifics not given)	Percent of unstable microsatellites	20	No
mSINGS	26 TCGA pan-cancer WXS and 298 pan-cancer gene panel samples	Read count differences between tumor sample and baseline normal	Fraction of unstable microsatellites	0.2	No
MANTIS	387 pan-cancer TCGA WXS samples	Absolute stepwise difference between tumor and normal read counts	Average aggregate instability	0.4	Yes
MSINGB	1432 pan-cancer TCGA WXS samples and 1055 pan-cancer non-TCGA WXS samples	NGBoost machine learning model based on somatic mutations	MSI status and probability of the classification	N/A (No score output)	No
PreMSIm	1383 pan-cancer TCGA RNA samples and 2006 gastric/colorectal microarray samples	K-nearest neighbors machine learning model based on gene expression	MSI status and probability of the classification	N/A (No score output)	No
MSIsensor-RNA	1428 pan-cancer TCGA RNA samples, 247 non-TCGA RNA samples, 1468 gastric/colorectal microarray samples, and 133 SC-RNA colorectal samples	Support vector machine learning classifier based on gene expression	MSI status and probability of the classification	N/A (No score output, but there are recommendations for feature selection thresholds)	No

A summary table of all the MSI tools used in this study. Whole genome sequencing and whole exome sequencing are abbreviated to WGS and WXS, respectively. Single-cell RNA sequencing was abbreviated to SC-RNA.

Baselines and microsatellite target sites were created for each tool based on their included reference genome scanner. For all these functions we supplied each tool with the same reference genome. Microsatellite bed files were generated using the default setting for each tool. Baseline files were created by giving each tool the same 20 randomly sampled WXS, WGS, or RNA normal BAM files. We chose 20 because this number has been suggested as the minimum number of required normal files by the authors of MSI-sensor pro (https://github.com/xjtu-omics/msisensor-pro/wiki/Frequently-Asked-Questions), and other researchers have used a similar number of normal samples to create an MSIngs baseline [38–40]. MSIsensor-RNA required an additional seven MSI-H samples to be included in the baseline. For MSIsensor2, MSINGB, and PreMSIm, we used the hg38 baseline files included with these tools as they either lacked a baseline generator [18], or their baseline generator could not be used successfully [21]. We also removed samples that had fewer than five microsatellite sites that passed the thresholds of each tool.

We first determined a threshold cut-off for each tool to classify a sample as MSI-H or MSS then measured performance for each tool. The recommended threshold cut-offs were 3.5 for MSIsensor, 20 for MSIsensor2, 0.2 for mSINGS, and 0.4 for MANTIS. Unfortunately, the authors of MSI-sensor pro do not provide a recommended threshold. Lastly, we used a threshold score of 20 for MSIsensor2 based on the author’s recommendations. Although the original MSIsensor publication recommended a threshold of 3.5, more recent publications have used a threshold of 10 [41,42]. We adjusted the threshold to 10 based on this. We also decided to use a threshold cut-off of 10 for MSIsensor-pro as it follows the same scoring system as its predecessor, MSIsensor.

We assessed how well each tool performed on each dataset by creating confusion matrices where the positive values were the MSI-H samples. Then we calculated precision, recall, specificity, and F1 score using equations outlined by Fawcett [43]. To view how these values change across different thresholds we created receiver operating characteristic (ROC) and precision-recall (PR) curves using the R packages MLeval [44], pROC [45], and caret [46]. We cross validated the ROC and PR curves using leave-one-out cross validation. All graphs were generated using R [47] and plotted with ggplot2 [48].

Lastly, we benchmarked the runtime and total memory used by each program on one random WXS, WGS, and RNA sample, where applicable, from TCGA. Runtime was measured using hyperfine [49] using the default settings except for the warmup parameter, which we set to 3, and the runtime of each tool was measured across 10 runs. We then measured total memory with the tool Massif available through Valgrind [50]. We set the pages-as-heap parameter to ‘yes’ which measures total heap memory used by a program, and the trace-children parameter to ‘yes’ which also measures the memory used by child processes. All benchmarks were done on a virtual machine running Ubuntu 20.04 LTS with 15 virtual CPUs and 65 gigabytes of RAM. While the virtual machine we created had multiple cores available, we chose to benchmark all tools in single-threaded mode. This is because only MANTIS, MSIsensor-pro, and MSIsensor-RNA were able to be run in multi-threaded mode, and we found that changing the multiple threads option either did not greatly improve the runtime of the program or caused it to crash before it could complete all 10 benchmarking runs. We chose not to include runtime or memory usage benchmarks for MSIsensor-RNA, PreMSIm, and MSINGB because they operate on prebuilt models making the time and memory used to run one sample negligible.

## Results

### MSI tools perform better on WXS than WGS samples

**Figure 1:**
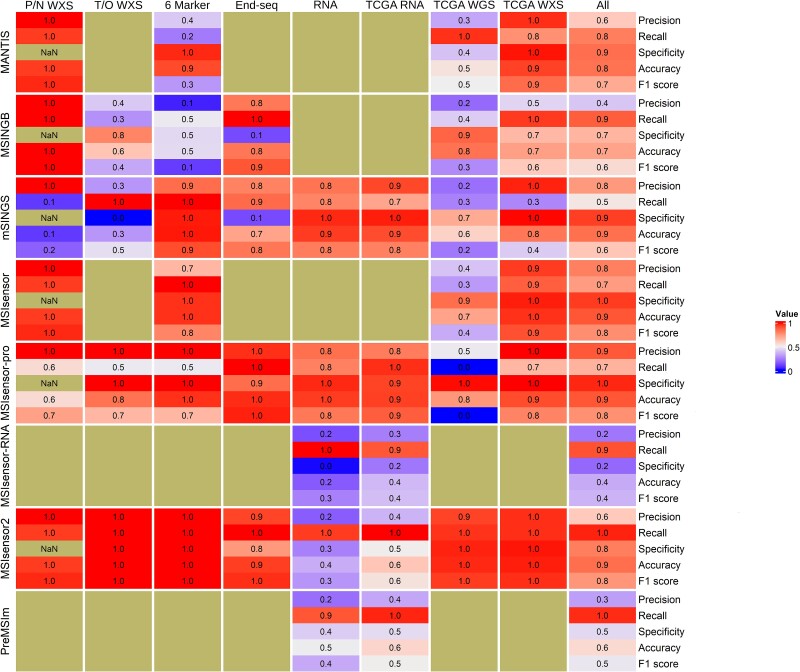
Performance metric heatmap. A heatmap showing all MSI tools and their performance across all datasets where a confusion matrix was created. The author recommended thresholds were used for each tool that provided one. Black tiles are NA values, and black and white striped tiles are instances where the metric could not be calculated. P/N WXS is the additional paired-normal whole exome sequencing dataset, T/O WXS is the additional tumor-only whole exome sequencing dataset. 6 marker stands for the 6-mononucleotide panel. TCGA WGS and WXS are the datasets comprised of whole exome and WGS data from The Cancer Genome Atlas, respectively. The ‘All’ column is the merged results for each tool.

**Figure 2:**
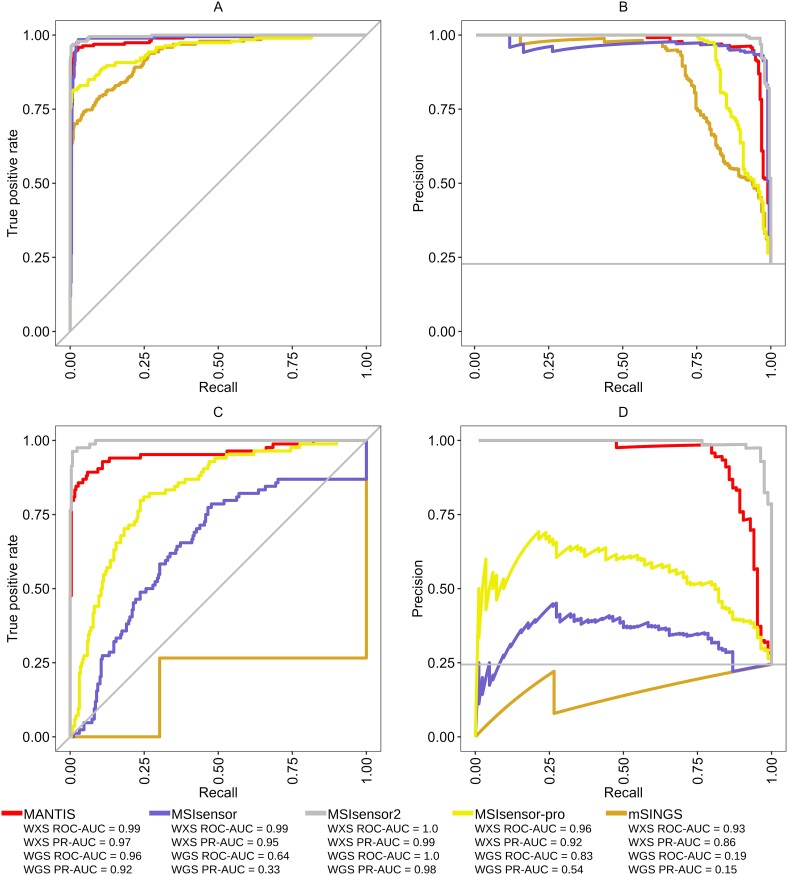
ROC and PR curves for all TCGA samples. All ROC curves and PR curves for TCGA WXS (A, B) and WGS (C, D) samples.

Using the published recommended settings most MSI tools performed better on WXS data than on WGS data (Tables 4 and 5, Fig. 1). The two exceptions were mSINGS and MSINGB, which had low performance metrics on the additional paired-normal and tumor-only WXS datasets (Table 5, Fig. 1). All the MSI tools showed good performance for TCGA WXS data, with again the exception of mSINGS, which had low recall and F1 scores (Table 4, Fig. 1). MSIsensor, MSIsensor-pro, MSIsensor2, and MANTIS all had high area under the curve (AUC) for both the ROC and PR curves (Fig. 2A, B). However, this was not always the case for the TCGA WGS data. Out of all MSI tools, only MSIsensor2 had high values for all performance metrics on WGS data (Table 4, Fig. 1). All other tools had one or more performance metrics substantially lower for WGS than for WXS data. The ROC and PR AUC values were also substantially lower for the WGS data than for the WXS data for all tools, except for MSIsensor2 and MANTIS (Fig. 2A, B, C, D). There were also large drop-offs in AUC when measured in ROC space versus PR space, implying tools might be missing more true positives (Fig. 2C, D). The most notable differences in ROC and PR AUC were seen with mSINGS on WXS data and with MSIsensor and MSIsensor-pro on WGS data (Fig. 2C, D).

**Table 4:** A confusion matrix for each MSI tool on all TCGA samples

Dataset	Tool	Number of samples	Number of filtered samples	TP	FP	TN	FN	Precision	Recall	F1 score	Accuracy	Specificity
TCGA WXS	MSIsensor-Pro	852	0	130	0	658	64	1.000	0.670	0.802	0.925	1.000
TCGA WXS	MSIsensor	852	0	172	9	649	22	0.950	0.887	0.917	0.964	0.986
TCGA WXS	MSIsensor2	852	0	188	6	652	6	0.969	0.969	0.969	0.986	0.991
TCGA WXS	mSINGS	849	3	54	1	654	140	0.982	0.278	0.434	0.834	0.998
TCGA WXS	MANTIS	852	0	150	5	653	44	0.968	0.773	0.860	0.942	0.992
TCGA WXS	MSINGB	852	0	185	221	437	9	0.456	0.954	0.617	0.730	0.664
TCGA WGS	MSIsensor-Pro	342	0	1	1	257	83	0.500	0.012	0.023	0.754	0.996
TCGA WGS	MSIsensor	342	0	27	38	220	57	0.415	0.321	0.362	0.722	0.853
TCGA WGS	MSIsensor2	339	3	78	5	253	3	0.940	0.963	0.951	0.976	0.981
TCGA WGS	mSINGS	324	18	21	73	172	58	0.223	0.266	0.243	0.596	0.702
TCGA WGS	MANTIS	341	1	81	151	106	3	0.349	0.964	0.513	0.548	0.412
TCGA WGS	MSINGB	226	0	7	26	184	9	0.212	0.438	0.286	0.845	0.876
TCGA RNA	MSIsensor-pro	825	0	181	39	599	6	0.823	0.968	0.889	0.945	0.939
TCGA RNA	MSIsensor2	825	0	187	306	332	0	0.379	1.000	0.550	0.629	0.520
TCGA RNA	mSINGS	750	75	121	9	572	48	0.931	0.716	0.809	0.924	0.985
TCGA RNA	MSIsensor-RNA	825	0	169	484	154	18	0.259	0.904	0.402	0.392	0.241
TCGA RNA	PreMSIm	825	0	183	338	300	4	0.351	0.979	0.517	0.585	0.470

We abbreviated whole genome sequencing to WGS, whole exome sequencing to WXS, true positive to TP, false positive to FP, true negative to TN, and false negative to FN. Although no MSINGB samples were filtered for TCGA WGS data, 116 samples took too long to process and were left out of the final analysis.

**Table 5:** Confusion matrix for all additional datasets

Dataset	Tool	Number of samples	Number of filtered samples	TP	FP	TN	FN	Precision	Recall	F1 score	Accuracy	Specificity
T/O WXS	MSIsensor-Pro	32	0	5	0	22	5	1.000	0.500	0.667	0.844	1.000
T/O WXS	MSIsensor2	32	0	10	0	22	0	1.000	1.000	1.000	1.000	1.000
T/O WXS	mSINGS	32	0	10	22	0	0	0.312	1.000	0.476	0.312	0.000
T/O WXS	MSINGB	30	2	3	4	16	7	0.429	0.300	0.353	0.633	0.800
P/N WXS	MSIsensor-Pro	21	0	12	0	0	9	1.000	0.571	0.727	0.571	NA
P/N WXS	MSIsensor	21	0	20	0	0	1	1.000	0.952	0.976	0.952	NA
P/N WXS	MSIsensor2	21	0	20	0	0	1	1.000	0.952	0.976	0.952	NA
P/N WXS	mSINGS	19	2	2	0	0	17	1.000	0.105	0.190	0.105	NA
P/N WXS	MANTIS	21	0	20	0	0	1	1.000	0.952	0.976	0.952	NA
P/N WXS	MSINGB	21	0	21	0	0	0	1.000	1.000	1.000	1.000	NA
End-seq	MSIsensor-Pro	36	0	29	1	6	0	0.967	1.000	0.983	0.972	0.857
End-seq	MSIsensor2	19	17	15	1	3	0	0.938	1.000	0.968	0.947	0.750
End-seq	mSINGS	36	0	25	6	1	4	0.806	0.862	0.833	0.722	0.143
End-seq	MSINGB	36	0	29	6	1	0	0.829	1.000	0.906	0.833	0.143
6 marker	MSIsensor-Pro	178	0	6	0	166	6	1.000	0.500	0.667	0.966	1.000
6 marker	MSIsensor	178	0	12	6	160	0	0.667	1.000	0.800	0.966	0.964
6 marker	MSIsensor2	174	4	12	0	162	0	1.000	1.000	1.000	1.000	1.000
6 marker	mSINGS	178	0	12	2	164	0	0.857	1.000	0.923	0.989	0.988
6 marker	MANTIS	178	0	3	4	162	9	0.429	0.250	0.316	0.927	0.976
6 marker	MSINGB	174	4	6	87	75	6	0.065	0.500	0.114	0.466	0.463
RNA	MSIsensor-Pro	142	3	17	3	118	4	0.850	0.810	0.829	0.951	0.975
RNA	MSIsensor2	142	3	20	89	32	1	0.183	0.952	0.308	0.366	0.264
RNA	mSINGS	141	4	16	3	118	4	0.842	0.800	0.821	0.950	0.975
RNA	PreMSIm	140	5	19	67	52	2	0.221	0.905	0.355	0.507	0.437
RNA	MSIsensor-RNA	143	2	21	118	4	0	0.151	1.000	0.262	0.175	0.033

A confusion matrix for each MSI tool on all additional samples. We abbreviated whole genome sequencing to WGS, whole exome sequencing to WXS, true positive to TP, false positive to FP, true negative to TN, and false negative to FN.

### MSI tools have widely different performance metrics depending on sequencing type and lack agreement on multiple sequencing types

To further evaluate tool performance, we merged the results of each tool across all datasets they could be tested on and calculated a confusion matrix (Supplementary Table 1, Fig. 1). Out of the paired-normal tools, MSIsensor had the highest performance metrics (Supplementary Table 1). MSIsensor2 and MSIsensor-pro both had the highest performance metrics when testing across all datasets., However, on the combined dataset, MSIsensor-pro had low recall (0.68) and MSIsensor2 had low precision (0.62). Both RNA sequencing tools had very high recall across all RNA sequencing datasets (0.91, 0.71), but all the other performance metrics were very low (Supplementary Table 1, Fig. 1).

**Figure 3:**
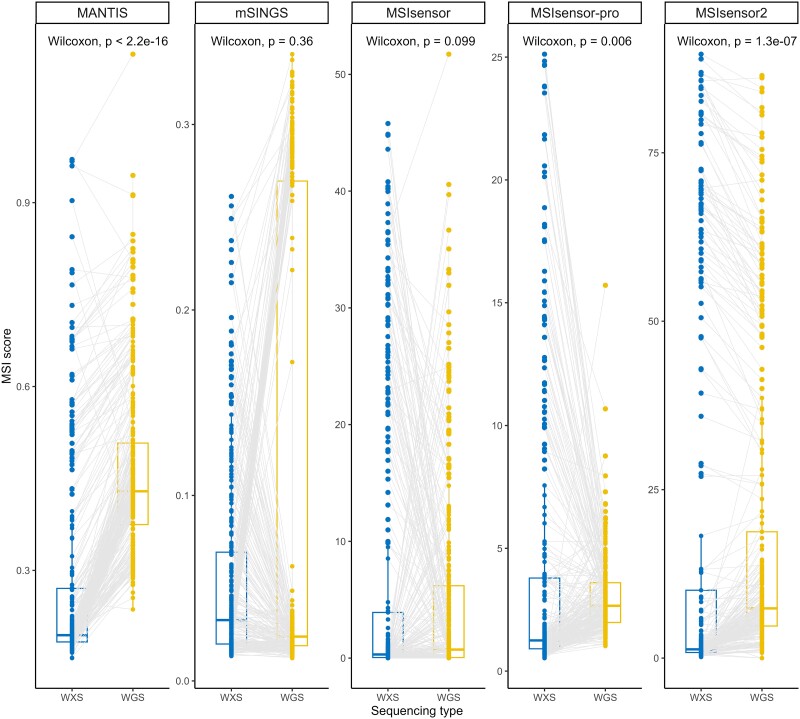
Boxplots of MSI scores for TCGA samples. Box plots of MSI scores for all TCGA samples that had both WGS and WXS samples. Gray lines were drawn between samples that have the same TCGA case ID. Paired Wilcox rank sum test values were calculated between the distribution of WXS and WGS scores for each tool.

To investigate the reduction in tool performance in WGS data relative to WXS data we compared MSI scores on 321 TCGA samples for which both types of data were available. In general, MSIsensor, MSIensor2, and MSIsensor-pro reported a higher proportion of unstable microsatellites for the WXS compared to the WGS data. In contrast, MANTIS and mSINGS scored samples higher for WGS versus WXS (Fig. 3). Interestingly, the tools that had high ROC and PR AUC scores on WGS samples, MANTIS and MSIsensor2, had significantly different MSI scores between WXS and WGS samples (Paired Wilcoxon rank sum test *P* = 2.2 × 10^−16^; *P* = 1.3 × 10^−7^; Fig. 3). This was also true to a lesser extent for MSIsensor-pro (*P* = 0.006), but it performed poorly on WGS data. MSIsensor and mSINGS did not have significantly different MSI scores between WGS and WXS samples (*P* > 0.05).

**Figure 4:**
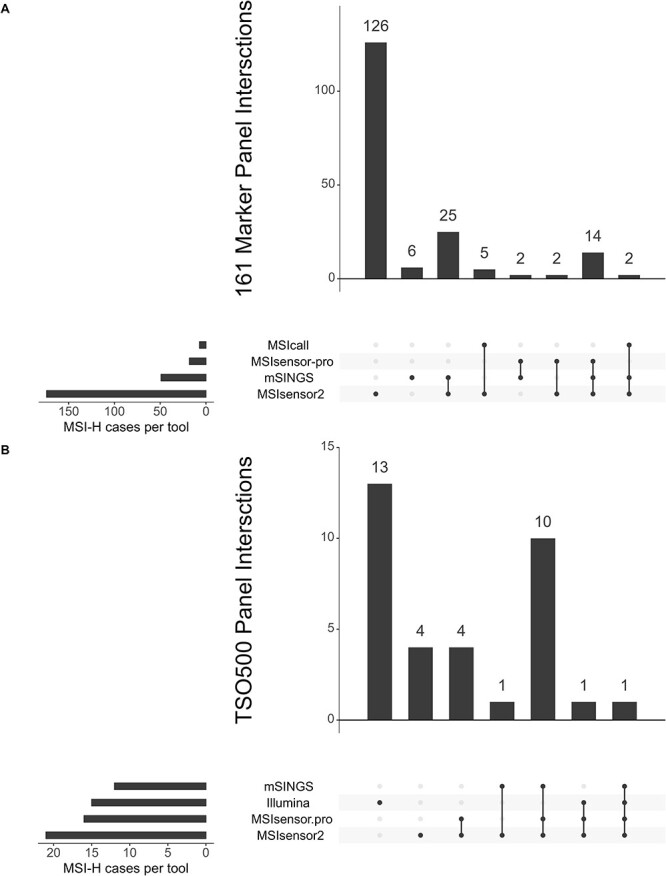
Upset plots for MSI tools on gene panel data. Upset plots showing intersections of MSI-H cases for each tool on 161-marker, Oncomine gene panel data (A) and TSO-500 panel data (B). Illumina and MSIcall are the MSI commercial for software results for the TSO-500 gene panel and 161-marker panel datasets, respectively. Connected dots show tools that agree on an MSI-H case whereas single dots are unique MSI-h cases for that tool. Vertical bars represent the total number of MSI-H cases that are agreed upon by one or more tools, and horizontal bars show the total number of MSI-H cases called by a tool.

When we applied the tools on gene panel data, we obtained strikingly inconsistent results (Fig. 4A, B). For both the TSO-500 data and 161-marker panel there was only one MSI-H sample in common between all methods tested (Fig. 4A, B). The largest overlaps were observed between MSIsensor2 and mSINGS for the 161-marker panel (25 cases), and between MSIsensor2, MSIsensor-pro, and mSINGS in the case of the TSO-500 data (10 cases). Surprisingly, MSIsensor2 identified 126 unique MSI-H cases in the Oncomine data (Fig. 4A). Overall, there was very little agreement between the evaluated tools and the commercial software included with the gene panels.

### Variation in performance by tool, threshold, and across data sets

**Figure 5:**
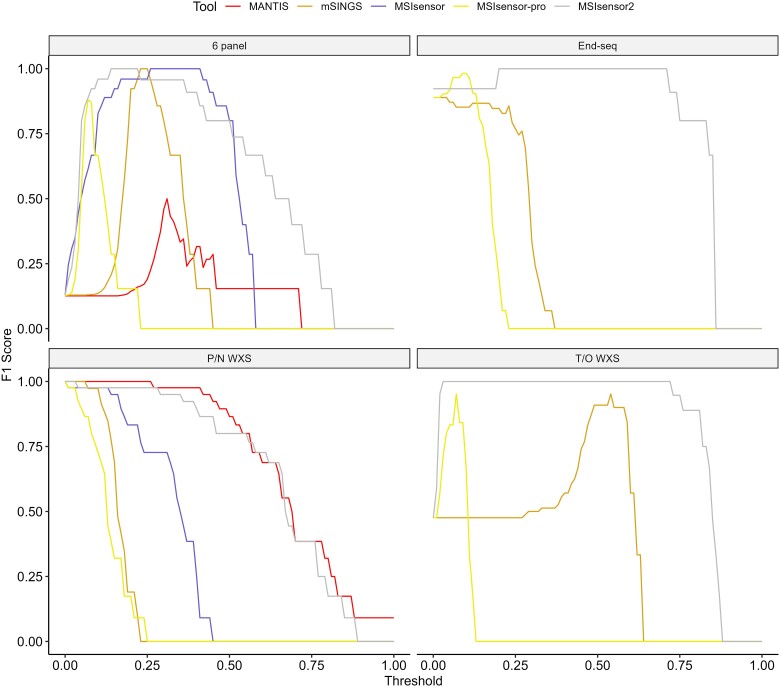
MSI tool performance on additional datasets by threshold. Graphs showing measures of performance (F1 scores) plotted across scaled MSI tool thresholds for each tool on additional datasets. These datasets included paired-normal whole exome sequencing data (P/N WXS), tumor-only whole exome sequencing data (T/O WXS), data from a 6-microsatellite marker panel (6 panel), and end-seq data. Thresholds were put on the same scale by converting MSI-sensor, MSI-sensor pro, and MSI-sensor 2 from percent to decimal.

To determine how well the author recommended WXS settings carry over to other sequencing types, we calculated F1 scores over varying thresholds for each tool across datasets that these tools would have no training exposure to (Fig. 5). All MSI tools were able to achieve a nearly perfect F1 score for all datasets if the correct optimal threshold for that dataset was used (aside from mSINGS on the 6-mononucleotide panel dataset). The only tool that demonstrated high F1 scores across a wide range of thresholds on all datasets was MSIsensor2. By contrast, MSIsensor-pro required very low and dataset-specific threshold values to achieve a high F1 score (Fig. 5). This result was also the case for accuracy, specificity, recall, and precision (Supplementary Figs 2–5).

The published thresholds for MSIsensor, mSINGS, and MANTIS on WXS provided good performance on the additional paired-normal WXS dataset (Fig. 5). However, achieving optimal performance, as measured by the F1 score, on the tumor only WXS dataset with mSINGS would require the use of a very different threshold (close to 0.5, compared to the recommended threshold of 0.2). Our results suggest that different thresholds may be required for use with different sequencing data types.

### MSI tools designed for DNA sequencing perform better than MSI tools designed for RNA sequencing on RNA sequencing datasets

We also evaluated the performance of tools designed for DNA when applied to RNA sequencing data. The two tools designed for use with RNA sequencing data, PreMSIm and MSIsensor-RNA, had low-performance metrics on the two RNA sequencing datasets used in our study (Fig. 1). While they could identify the MSI-H cases in the dataset well (recall ≥0.9), all other performance metrics were low (Tables 4 and 5). Interestingly, two of the tools designed for DNA sequencing, MSIsensor-pro and mSINGS had high F1 scores on both the TCGA RNA sequencing data (0.81–0.90) (Table 4) and on the additional bulk RNA sequencing dataset (0.83–0.84) (Table 5). Similar to the RNA sequencing tools, MSIsensor2 had high recall scores (0.9–1.0) but very low precision scores (0.18–0.37) (Tables 4 and 5). When tested across many thresholds with ROC and PR curves, all three DNA sequencing tools had high values for both ROC-AUC (0.97–.99) and PR-AUC (0.92–0.99) (Supplementary Fig. 1A, B).

### Tools vary greatly in runtime and memory usage

Lastly, we measured average runtime across 10 runs and total memory usage for all tools. We found that there were substantial differences in runtime and memory usage between tools (Supplementary Fig. 6, Supplementary Tables 2 and 3). Most notably, MSIsensor2 and MSIsensor-pro were much faster than the other tools, taking an average of 115 and 90.86 seconds, respectively, to process a WGS sample. While the other tools took upwards of 3800 seconds to process both WXS and WGS samples, MANTIS had the worst runtime, taking an average of 14 111 seconds to process a WGS sample and 6410 seconds to process a WXS sample. Although it had the worst average runtime, MANTIS was by far the most efficient in terms of memory usage, using only 23.34 megabytes to process a WGS sample (Supplementary Table 3). All other tools had similar memory usage to one another, except for mSINGS, which required over 30 gigabytes of memory to process either a WGS or WXS sample.

## Discussion

We chose eight MSI tools to benchmark. Out of the many tools that are available, these eight have common pipelines making them easy to compare and run in a parallel workflow. Many other MSI NGS methods exist, but we found they were either lacking a downloadable tool [23,51,52], they were deprecated [53], or they did not output an MSI score or status [54]. While we chose to add RNA sequencing and two tools that predict MSI status based on expression, we did so because the other tools used in our study were compatible with these data types. We chose not to include additional, multi-omic approaches [13, 55, 56] as the data they require is difficult to obtain in large numbers from publicly available repositories.

Although the developers of several MSI tools have reported very high performance metrics, we found that performance can be severely affected by sequencing type and the choice of threshold used to classify samples as MSI-H. Many MSI tools were originally tested using WXS data from TCGA and, indeed, on these data tools often achieved high performance; however, they had worse performance metrics on WGS data (Figs 1, 2A, B, C, D). Further, MSI tools scored the same TCGA individuals differently with WXS compared to WGS data, with some having significantly different MSI scores (Fig. 3). There was also an overall lack of tool agreement on MSI-H status with gene panel data, and some tools required specific thresholds for different sequencing types to achieve a high F1 score. Lastly, we found tools designed for RNA sequencing data not only had low performance metrics on RNA sequencing datasets, but that some tools developed for DNA sequencing data had higher performance metrics on these data.

The authors of multiple tools have suggested they can be used with a variety of sequencing types [17–20], but only one includes more than one sequencing type in its original publication [18]. The lack of validation on different sequencing methods can be problematic as we have shown that some of the tools are not suitable for use with WGS. This is highlighted primarily by the reduction in ROC-AUC and PR-AUC when compared to the TCGA WXS results. The worst performer on WGS data, mSINGS, had an abnormal looking ROC curve due to its MSI scores being noninformative for the data. The horizontal and vertical bars seen, as opposed to usual jagged cut-points, are the result of no increase in true positive rate despite change in threshold and sudden increase in true positive rate, respectively.

The differences in tool performance on WGS and WXS datasets are likely attributable to the number of microsatellites included in the analysis and the overall coverage of microsatellites. The tradeoff between these two sequencing strategies is that many more microsatellites are sequenced with WGS, but the sites sequenced with WXS are at a much higher coverage. It could be the case that simply including fewer microsatellites in the analysis while sequencing them at a much higher coverage is a better strategy for detecting MSI. Another factor to consider is that exonic regions contain a higher proportion of trinucleotide and hexanucleotide repeat types, whereas intronic regions contain more of the other repeat types [57]. There is the possibility that microsatellites in exonic regions are more indicative of MSI than intronic regions, but the current paradigm suggests mononucleotide and dinucleotide microsatellites are the most sensitive for MSI detection [58]. From our results, two tools, MANTIS and MSIsensor2 had high ROC and PR AUC scores for both WGS and WXS datasets. MANTIS uses a more complex aggregate scoring approach that calculates normalized distance values as opposed to the locus-by-locus strategy of the other MSI tools. Alongside its aggregate approach to determining MSI status, it includes additional filtering steps, which could potentially help explain its success with WGS data.

While MSIsensor2 performed well on TCGA WGS and WXS data, it failed to process most End-seq samples because no microsatellite sites passed the filter thresholds. In principle, End-seq should be a compatible sequencing method with this tool because it is a genome-wide sampling scheme like WGS that works well with repair-deficient genotypes [59] and it has been shown to have similar read depth to WXS with upwards of 100x coverage [34]. However, the poor performance of MSIsensor2 on this sequencing type suggests caution is needed when using MSI tools on novel datasets.

Aside from the End-seq data, MSIsensor2 showed the best performance across all other datasets. This is surprising, as MSIsensor2 is unpublished and seems to only have been used by the GDC for ‘bioinformatics-derived’ MSI status (https://docs.gdc.cancer.gov/Data/Bioinformatics_Pipelines/DNA_Seq_Variant_Calling_Pipeline/). Unfortunately, there is very limited information on the machine learning algorithm used by MSIsensor2 and no references are provided to the samples that have been used to train the hg38 models they provide [18]. Because of this, the high performance metrics seen in our results should be treated with caution. Another striking result we found with MSIsensor2 was the number of unique MSI-H cases identified in the 161-marker gene panel data. The highest estimates of MSI status come from colorectal cancers where occurrence is seen in ~15% of cancers [60]. Therefore, the number of MSI-H cases reported by MSIsensor2 is unlikely to be close to the correct value, as it classified ~74% of these 191 samples as MSI-H. Although the results obtained by MSIsensor2 on all other datasets were promising, this aberrant result is concerning and suggests that further testing on additional data types may be required.

Importantly, this benchmarking analysis highlights the risks of establishing recommended settings for MSI tools based on relatively narrow training cohorts. For example, MSIsensor established its recommended MSI-H threshold based on only 242 endometrial samples [11] (Table 3). While we were able to replicate the ROC-AUC values of these tools on TCGA WXS data, we found the optimal F1 score impossible or difficult to achieve with recommended threshold settings. This has led to situations where researchers try to account for this by simply picking the optimal threshold for their specific dataset [61], or where researchers report difficulty in determining a clear threshold for MSI scores at all [62]. Ideally, thresholds to determine MSI-H status for a given sequencing type should be validated with a large and diverse cohort as was done for MSI-sensor on MSK-impact gene panels [63], which serves as a good example of how to integrate these tools into a precision medicine setting.

## Conclusion

Several computational tools have been developed to classify NGS samples as MSI-H or MSS and they have generally been reported to achieve high performance. However, we have found that there are potentially serious issues affecting the reliability of these tools, particularly when applied across diverse sequencing data types. Notably, there was a large drop in performance on WGS relative to WXS samples. Tools were also shown to lack agreement on gene panel samples both with one another and with commercially available software, and that optimal thresholds can change with sequencing type. Lastly, we showed that tools designed for use with RNA sequencing data had poor performance metrics on bulk RNA test datasets and were even outperformed by some MSI tools meant to be used with DNA sequencing data.

Two MSI tools stood out from the others, MSIsensor2 and MANTIS. Both performed very well across most datasets, but the methodology of MSIsensor2 has yet to be published and there is currently no adequate information available on the algorithm it uses. When looking across all datasets the performance metrics of both tools were much lower. It also could not process most of the End-seq samples and classified a very large and implausible number of samples as MSI-H in a 161-marker panel dataset. MANTIS on the other hand did not achieve high performance metrics on a 6-marker panel dataset, still requires a paired-normal sample, and has a slow runtime in comparison to other MSI tools, limiting its applicability and rendering it hard to integrate into some genome profiling pipelines.

We have shown evidence that when tested outside their optimal settings, MSI NGS tools can have difficulty replicating the performance originally seen on TCGA WXS data. This problem is further compounded by a lack of clear best practice settings, and until more MSI NGS datasets are publicly available for additional testing, it is difficult to validate the applicability of MSI tools on additional sequencing types. Altogether, this research highlights several concerns relating to MSI tools used with NGS data and suggests that, despite the high-performance metrics reported for several tools, there remains a need for a rigorously evaluated tool that can be reliably applied to a wide range of sequencing data types.

**Key Points**

- Tools designed to determine MSI status performed worse on whole genome sequencing data than on whole exome sequencing data.
- Two tools, MSI-sensor 2 and MANTIS, had excellent performance across all datasets, but each comes with its own drawbacks.
- Optimal choice of MSI-H threshold depends strongly on the data type.
- Tools that infer the MSI status based on read counts performed better on RNA sequencing data than tools that use gene count matrices to infer the MSI status.

## Supplementary Material

supplementary_files_bbae390

supplementary_figure2_hd_bbae390

sup_fig6_hd_bbae390

## Data Availability

All data used in this research are available from the GDC data portal (https://portal.gdc.cancer.gov/) or from the SRA archive (https://www.ncbi.nlm.nih.gov/sra). All related project ID’s or accession numbers are included in Tables 1 and 2 of this research article. The code used to generate results, figures, and tables in this research article are available from our GitHub (https://github.com/harrison-anth/benchmark_msi).
