# Supplementary material for: Performance assessment of computational tools to detect microsatellite instability
Source: Brief Bioinform. 2024 Aug 12;25(5):bbae390. doi: 10.1093/bib/bbae390 (PMC11317526; doi:10.1093/bib/bbae390)
Supplement: supplementary_files_bbae390 [file supplementary_files_bbae390.zip › all_datasets_merged_table_minor_revision.docx]

| Dataset | Tool | Number of samples | TP | FP | TN | FN | Precision | Recall | F1 score | Accuracy | Specificity |
| --- | --- | --- | --- | --- | --- | --- | --- | --- | --- | --- | --- |
| All | MSIsensor-pro | 2286 | 364 | 41 | 1708 | 173 | 0.899 | 0.678 | 0.773 | 0.906 | 0.977 |
| All | MSIsensor2 | 2262 | 510 | 318 | 1424 | 10 | 0.616 | 0.981 | 0.757 | 0.855 | 0.817 |
| All | mSINGS | 2938 | 366 | 122 | 2135 | 315 | 0.750 | 0.537 | 0.626 | 0.851 | 0.946 |
| All | MSINGB | 1343 | 251 | 346 | 715 | 31 | 0.420 | 0.890 | 0.571 | 0.719 | 0.674 |
| All | MSIsensor | 1393 | 231 | 53 | 1029 | 80 | 0.813 | 0.743 | 0.776 | 0.905 | 0.951 |
| All | MANTIS | 1392 | 254 | 160 | 921 | 57 | 0.614 | 0.817 | 0.701 | 0.844 | 0.852 |
| All | MSIsensor-RNA | 968 | 190 | 602 | 158 | 18 | 0.240 | 0.913 | 0.380 | 0.360 | 0.208 |
| All | PreMSIm | 965 | 202 | 405 | 352 | 6 | 0.333 | 0.971 | 0.496 | 0.574 | 0.465 |

Supplementary Table 1: A confusion matrix for each MSI tool on all applicable datasets. All possible MSI score results for each tool were merged to create this confusion matrix. We abbreviated, true positive to TP, false positive to FP, true negative to TN, and false negative to FN.
