# Supplementary material for: Performance assessment of computational tools to detect microsatellite instability
Source: Brief Bioinform. 2024 Aug 12;25(5):bbae390. doi: 10.1093/bib/bbae390 (PMC11317526; doi:10.1093/bib/bbae390)
Supplement: supplementary_files_bbae390 [file supplementary_files_bbae390.zip › supplementary_table3.docx]

| Tool | Run 1 | Run 2 | Run 3 | Run 4 | Run 5 | Run 6 | Run 7 | Run 8 | Run 9 | Run 10 | Average | Total memory (mb) |
| --- | --- | --- | --- | --- | --- | --- | --- | --- | --- | --- | --- | --- |
| MSIsensor | 3835.61 | 3846.46 | 3825.74 | 3824.52 | 4030.02 | 3829.61 | 3839.94 | 3827.47 | 3981.99 | 3827.30 | 3866.87 | 1035.74 |
| MSIsensor-pro | 90.84 | 91.09 | 90.52 | 90.42 | 93.33 | 91.44 | 90.10 | 90.09 | 89.24 | 91.50 | 90.86 | 709.92 |
| MSIsensor2 | 120.03 | 114.33 | 113.06 | 114.83 | 119.52 | 114.11 | 112.81 | 114.08 | 113.78 | 113.44 | 114.99 | 275.19 |
| mSINGS | 6011.43 | 6021.07 | 6036.45 | 5986.41 | 6005.93 | 5965.60 | 5982.29 | 5986.75 | 5934.27 | 5970.93 | 5990.12 | 34138.50 |
| MANTIS | 13975.40 | 13984.15 | 14001.04 | 14096 | 14313.02 | 14214.13 | 14267.36 | 14073.13 | 14082.40 | 14104.62 | 14111.13 | 23.34 |

Supplementary Table 3: The run times for each tool on a random whole genome sequencing, TCGA sample. Each run time is measured in seconds, and the average is measured from the 10 runs. Total memory is measured in megabytes and represents the total heap memory used by a tool for a single run.
