# Supplementary figures and images for: Performance assessment of computational tools to detect microsatellite instability
Source: Brief Bioinform. 2024 Aug 12;25(5):bbae390. doi: 10.1093/bib/bbae390 (PMC11317526; doi:10.1093/bib/bbae390)

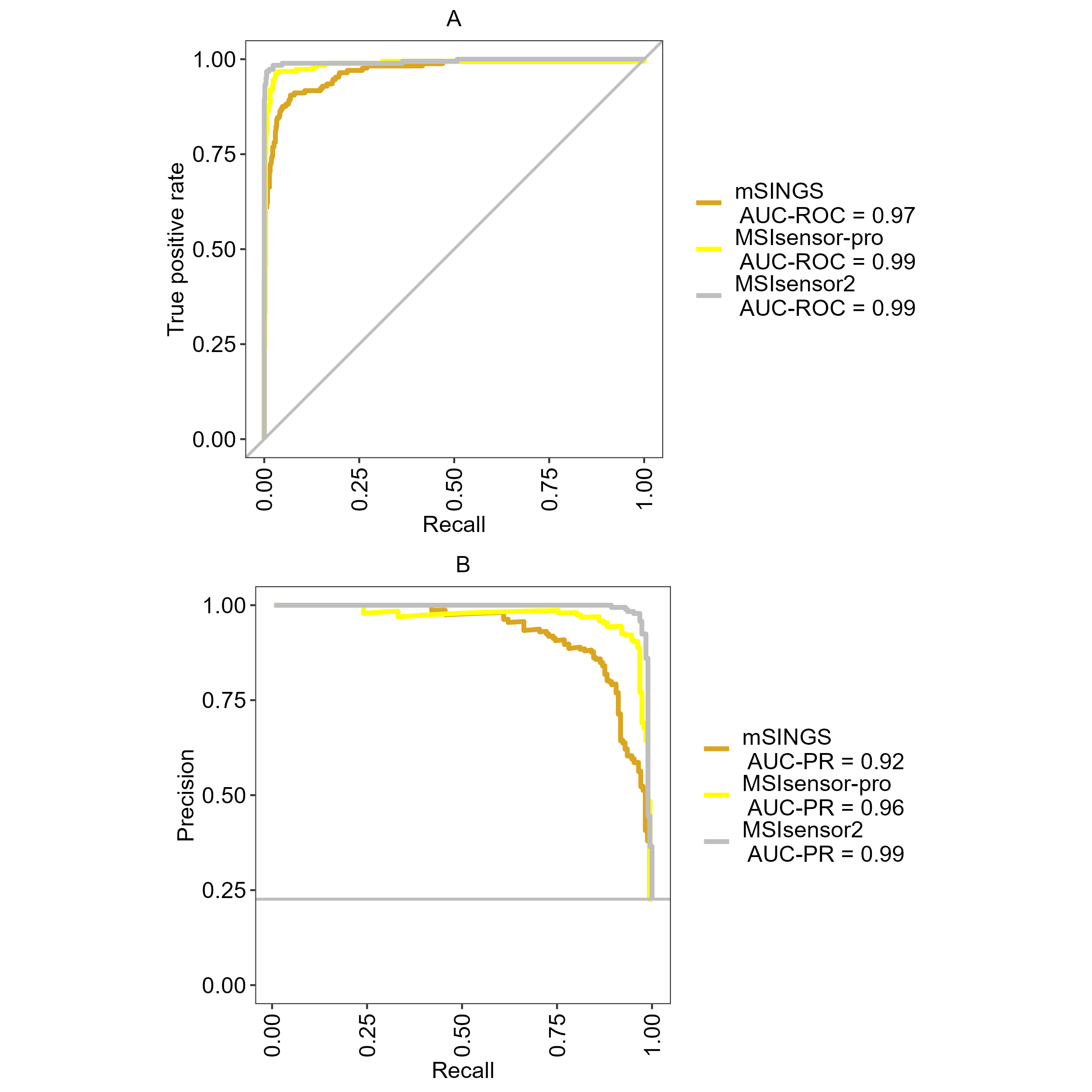

Supplement: supplementary_files_bbae390 [file supplementary_files_bbae390.zip › supplementary_figure1_hd_take2.tiff]

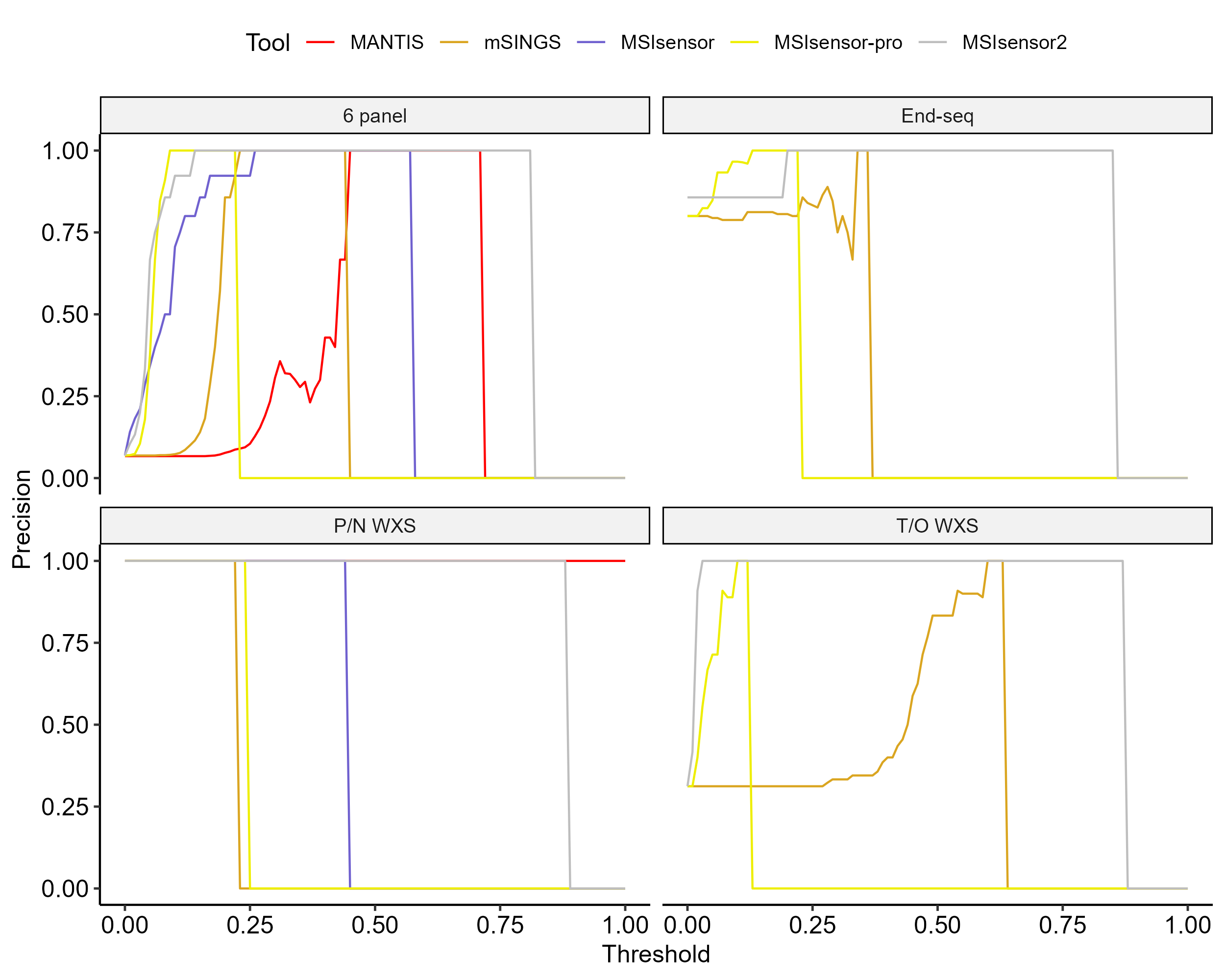

Supplement: supplementary_files_bbae390 [file supplementary_files_bbae390.zip › supplementary_figure2_hd.tiff]

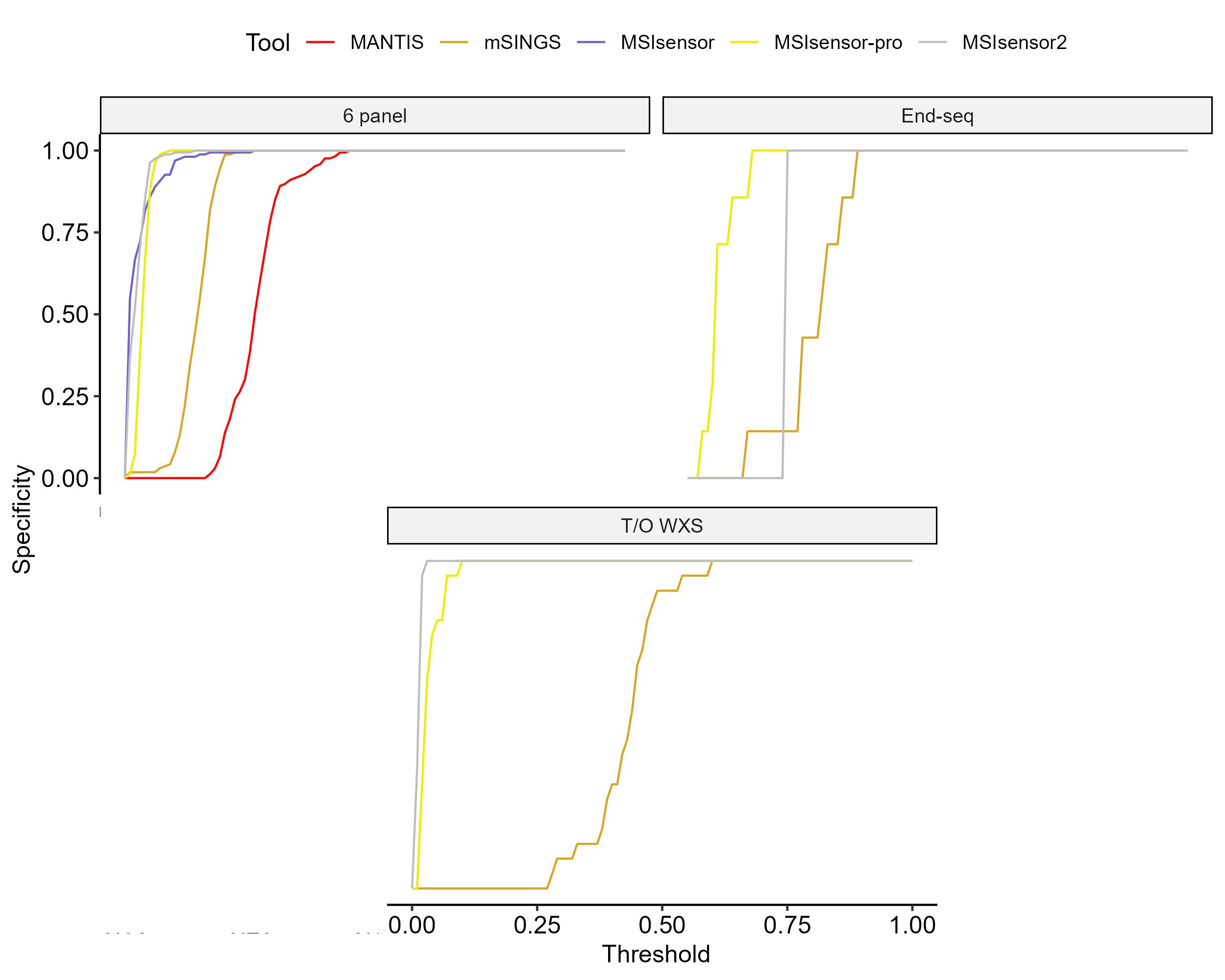

Supplement: supplementary_files_bbae390 [file supplementary_files_bbae390.zip › supplementary_figure3_hd_cropped_take2.tiff]

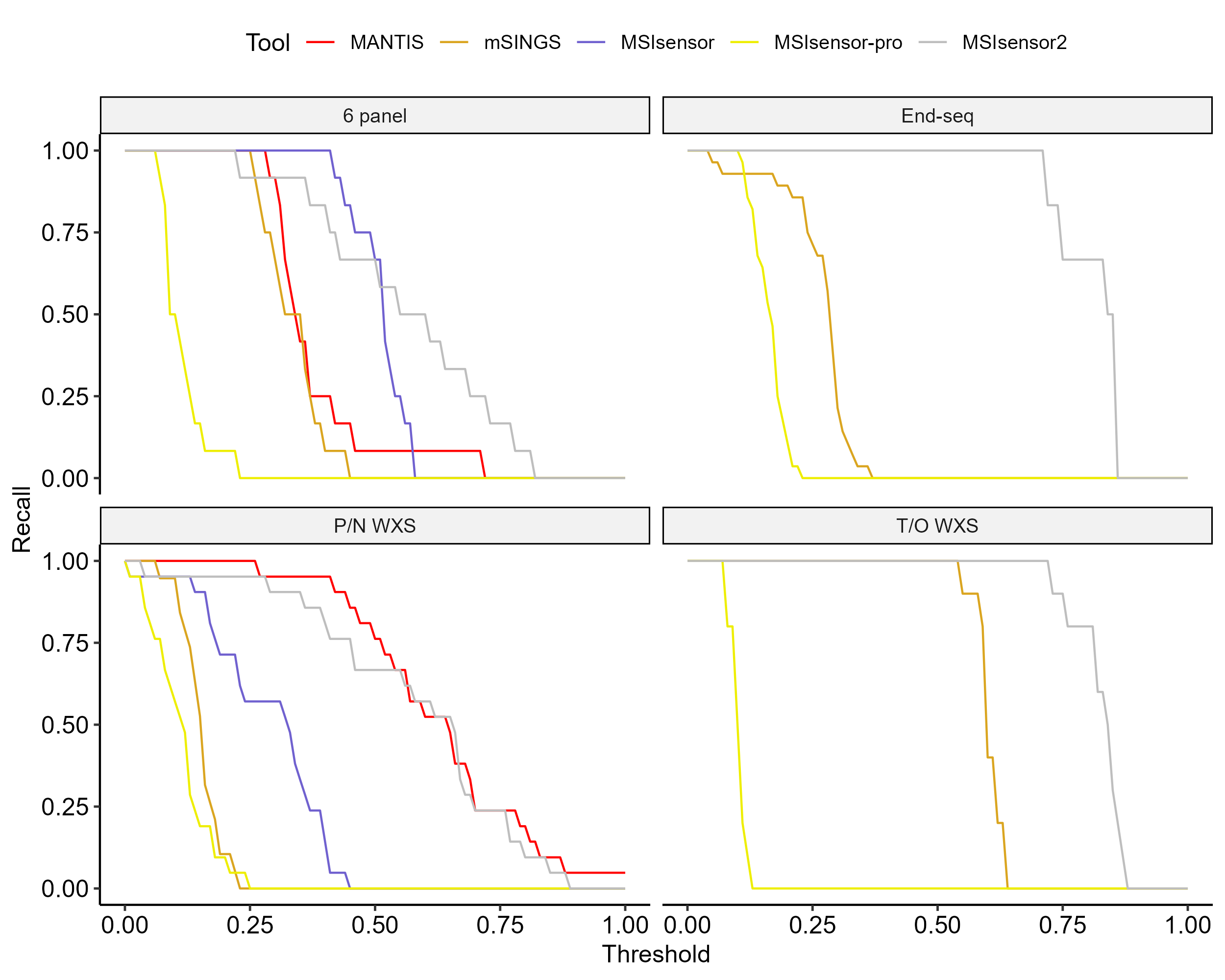

Supplement: supplementary_files_bbae390 [file supplementary_files_bbae390.zip › supplementary_figure4_hd.tiff]

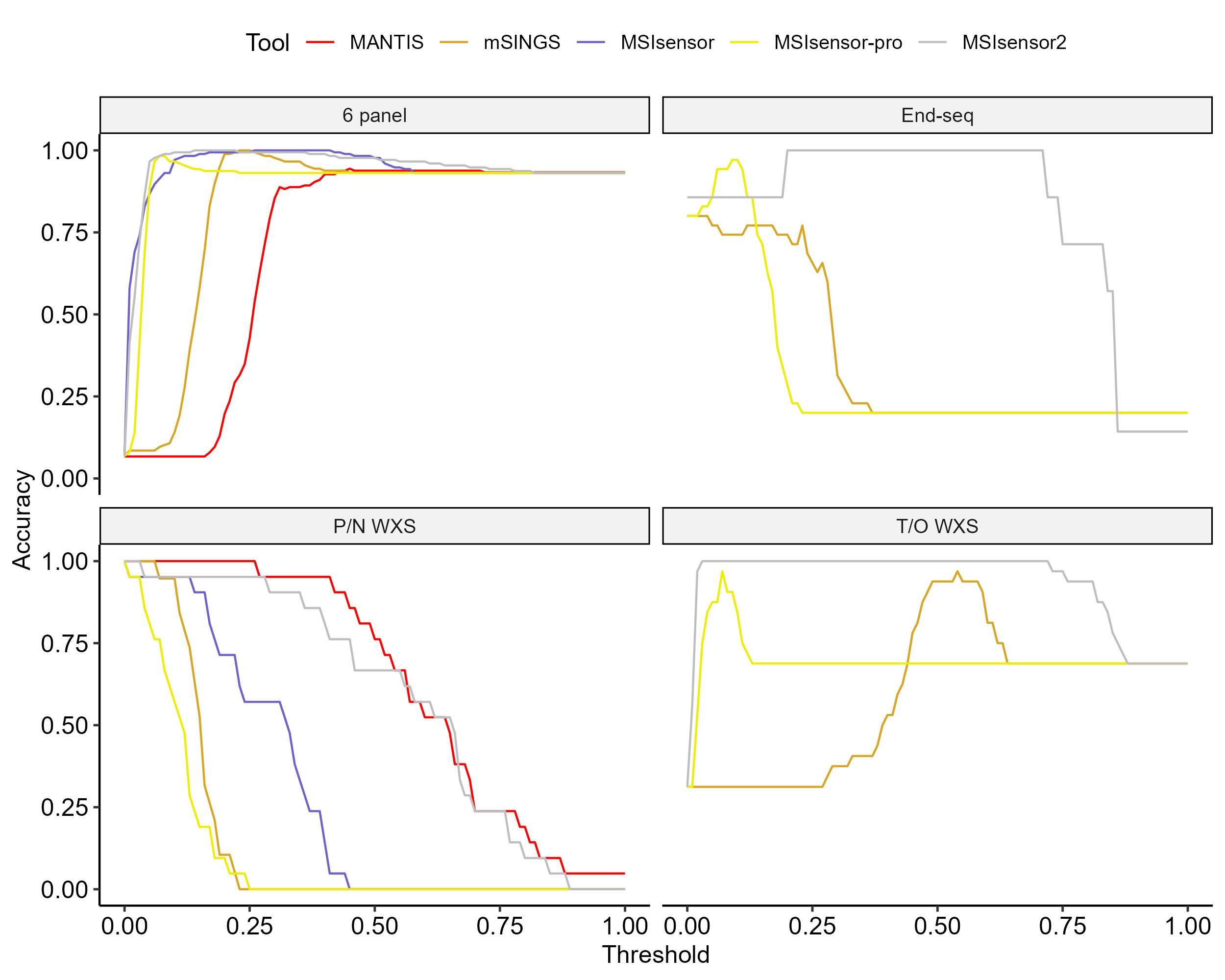

Supplement: supplementary_figure2_hd_bbae390 [file supplementary_figure2_hd_bbae390.jpeg]

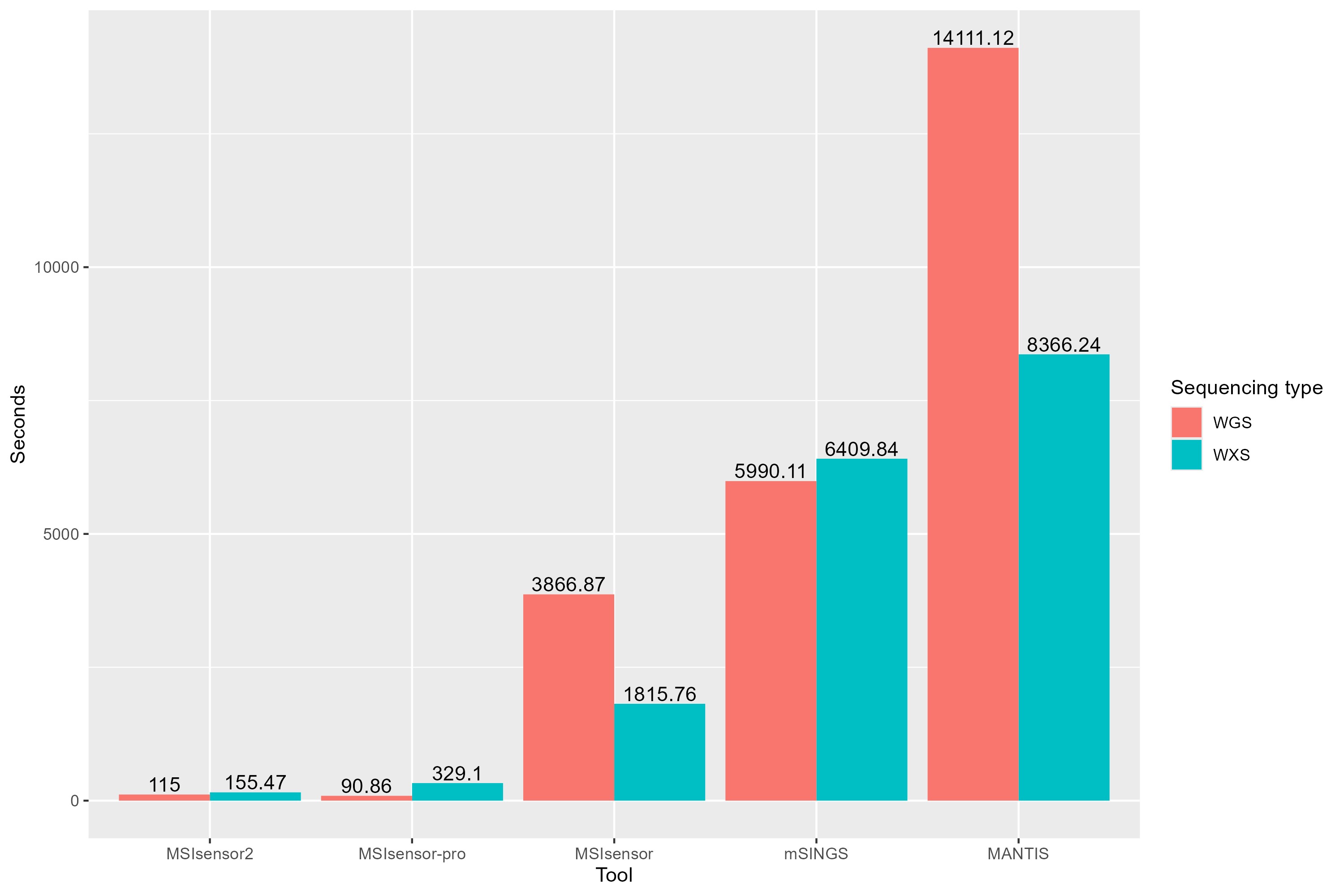

Supplement: sup_fig6_hd_bbae390 [file sup_fig6_hd_bbae390.jpeg]
